# *tert*-Butyl 5-(4-methoxy­phen­yl)-1-methyl-2-oxopyrrolidin-3-yl carbonate

**DOI:** 10.1107/S1600536808005527

**Published:** 2008-03-05

**Authors:** M. Fazli Mohammat, Zurina Shaameri, A. Sazali Hamzah, Hoong-Kun Fun, Suchada Chantrapromma

**Affiliations:** a Institute of Science, Universiti Teknologi MARA, 40450 Shah Alam, Selangor, Malaysia; b X-ray Crystallography Unit, School of Physics, Universiti Sains Malaysia, 11800 USM, Penang, Malaysia; c Department of Chemistry, Faculty of Science, Prince of Songkla University, Hat-Yai, Songkhla 90112, Thailand

## Abstract

In the title compound, C_17_H_23_NO_5_, the pyrrolidinone ring is in an envelope conformation. The *tert*-butyl carbonate and 4-methoxy­phenyl groups are arranged on the same side of the pyrrolidinone ring. The meth­oxy group is coplanar with the attached benzene ring. The mol­ecules are linked into chains along the *b* axis *via* C—H⋯O hydrogen bonds.

## Related literature

For bond-length data, see: Allen *et al.* (1987 ▶). For ring conformations, see: Cremer & Pople (1975 ▶). For the biological properties of pyrrolidine alkaloids, see: Iida *et al.* (1986 ▶); Matkhalikova *et al.* (1969 ▶); Reddy & Rao (2006 ▶); Royles (1996 ▶). For syntheses of compounds containing a tetra­mic acid ring, see: Chandrasekhar *et al.* (2005 ▶, 2006 ▶); Gurjar *et al.* (2006 ▶); Yoda *et al.* (1996 ▶). For a related structure, see: Mohammat *et al.* (2008 ▶).
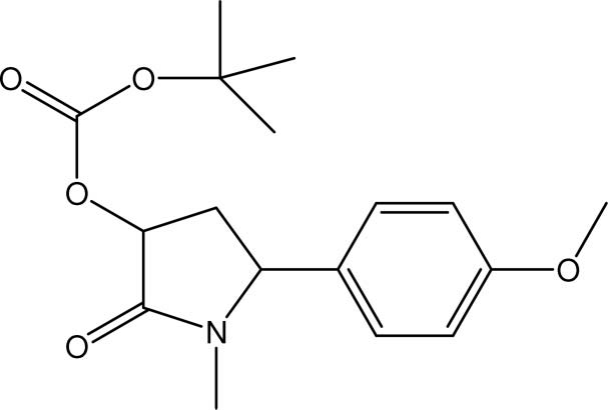


## Experimental

### 

#### Crystal data

C_17_H_23_NO_5_
*M* *_r_* = 321.36
Monoclinic, 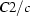

*a* = 23.9157 (4) Å
*b* = 6.2788 (1) Å
*c* = 24.1224 (4) Å
β = 101.971 (1)°
*V* = 3543.49 (10) Å^3^
*Z* = 8
Mo *K*α radiation
μ = 0.09 mm^−1^
*T* = 100.0 (1) K
0.49 × 0.18 × 0.16 mm

#### Data collection

Bruker SMART APEXII CCD area-detector diffractometer
Absorption correction: multi-scan (*SADABS*; Bruker, 2005 ▶) *T*_min_ = 0.958, *T*_max_ = 0.986
21759 measured reflections
5155 independent reflections
3632 reflections with *I* > 2σ(*I*)
*R*_int_ = 0.035

#### Refinement

*R*[*F* ^2^ > 2σ(*F* ^2^)] = 0.048
*wR*(*F* ^2^) = 0.125
*S* = 1.09
5155 reflections
213 parameters
H-atom parameters constrained
Δρ_max_ = 0.30 e Å^−3^
Δρ_min_ = −0.27 e Å^−3^

### 

Data collection: *APEX2* (Bruker, 2005 ▶); cell refinement: *APEX2*; data reduction: *SAINT* (Bruker, 2005 ▶); program(s) used to solve structure: *SHELXTL* (Sheldrick, 2008 ▶); program(s) used to refine structure: *SHELXTL*; molecular graphics: *SHELXTL*; software used to prepare material for publication: *SHELXTL* and *PLATON* (Spek, 2003 ▶).

## Supplementary Material

Crystal structure: contains datablocks global, I. DOI: 10.1107/S1600536808005527/ci2562sup1.cif

Structure factors: contains datablocks I. DOI: 10.1107/S1600536808005527/ci2562Isup2.hkl

Additional supplementary materials:  crystallographic information; 3D view; checkCIF report

## Figures and Tables

**Table 1:** Hydrogen-bond geometry (Å, °)

*D*—H⋯*A*	*D*—H	H⋯*A*	*D*⋯*A*	*D*—H⋯*A*
C2—H2⋯O1^i^	1.00	2.35	2.9633 (15)	119
C4—H4⋯O1^ii^	1.00	2.56	3.5238 (15)	162
C11—H11*A*⋯O1	0.98	2.47	2.8652 (16)	104
C15—H15*A*⋯O3	0.98	2.45	3.011 (3)	116
C16—H16*B*⋯O3	0.98	2.44	2.9904 (19)	115
C17—H17*A*⋯O3^iii^	0.98	2.38	3.324 (2)	161

Symmetry codes: (i) 
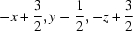
; (ii) 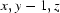; (iii) 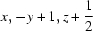.

**Fig. 1.**
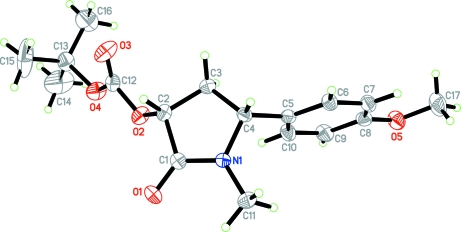
The molecular structure of the title compound, showing 50% probability displacement ellipsoids and the atomic numbering.

**Fig. 2.**
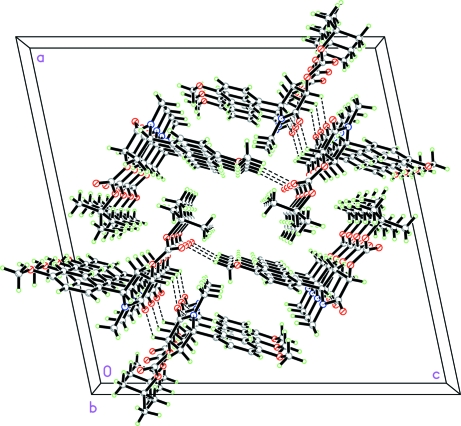
The crystal packing of the title compound, viewed approximately along the b axis. Hydrogen bonds are shown as dashed lines.

## supplementary crystallographic information

### Comment

Chiral polyhydroxy alkaloids show remarkable biological properties. Among these, pyrrolidine alkaloids carrying an aromatic substituent on the ring are of a rare class found in nature (Reddy & Rao, 2006). The title compound, C_17_H_23_NO_5_, can act as an essential intermediate in the synthesis of such a hydroxyl alkaloid unit (Chandrasekhar *et al.*, 2005; 2006; Gurjar *et al.*, 2006; Yoda *et al.*, 1996), which eventually can be used as a template in multi-step syntheses of natural products such as codonopsinine isolated from *Codonopsis clematidae*. Codonopsinine exhibits antibiotic and hypotensive activities without affecting the central nervous system (Matkhalikova *et al.*, 1969). We have synthesized the title compound and its structure is reported here.

The molecular structure of the title compound is shown in Fig. 1. The pyrrolidinone ring adopts an envelope conformation, with atom C3 displaced from the C1/C2/C4/N1 plane by 0.310 (2) Å; the puckering parameters (Cremer & Pople, 1975) are Q = 0.194 (1) Å and φ = 111.1 (4)°. The methoxy group is coplanar with the benzene ring as indicated by the torsion angle C17–O5–C8–C7 of -2.06 (19)°. In the *tert*-butylcarbonate moiety, atoms C12, C13, C14, O2, O3 and O4 lie on the same plane, with O4 deviating by a maximum of 0.019 (1) Å. All bond lengths and angles show normal values (Allen *et al.*, 1987) and are comparable with those observed in a related structure (Mohammat *et al.*, 2008).

Weak C—H···O intramolecular interactions are observed in the molecular structure. In the crystal packing (Fig. 2), the molecules are linked into chains along the *b* axis *via* C2—H2···O1^i^, C4—H4···O1^ii^ and C17—H17A···O3^iii^ hydrogen bonds (Table 1).

### Experimental

Equimolar amount of diethyloxalacetate sodium salt (20.00 g, 95.2 mmol), anisaldehyde (11.60 ml, 95.2 mmol) and methylamine (11.74 ml, 95.2 mmol) in ethanol (200 ml) was refluxed to obtain an *α*,*β*-diketo ester (7.78 g, 28%). Diethoxycarbonylation of this *α*,*β*-diketo ester (2.62 g, 8.94 mmol) was then successfully carried out by refluxing in 10% HCl solution to give a basic pyrrolidinone ring skeleton (0.86 g, 44%). Reduction of this diketone (0.32 g, 1.46 mmol) was carried out in sodium borohydride/methanol at 273 K to give the hydroxy keto amide (0.29 g, 92%). Protection of the hydroxyl group (0.29 g, 1.3 mmol) was successfully carried out using *tert*-butoxycarbonyl (Boc_2_O), and 4-dimethylaminopyridine (DMAP) in tetrahydrofuran (THF) *via* stirring at room temperature for 24 h to obtain the title compound in 76% yield (0.31 g). Colourless block-shaped single crystals suitable for *X*-ray structure determination were obtained by slow evaporation of an ethyl acetate-petroleum ether (1:1 *v*/*v*) solution after several days.

### Refinement

H atoms were placed in calculated positions with C—H = 0.95 Å (aromatic), 0.98 Å (CH_3_), 0.99 Å (CH_2_) and 1.00 Å (CH), and with *U*_iso_ = 1.5*U*_eq_(C) for CH_3_ atoms and 1.2*U*_eq_(C) for other H atoms. A rotating group model was used for methyl groups.

### Crystal data

**Table:**

C_17_H_23_NO_5_	*F*_000_ = 1376
*M**_r_* = 321.36	*D*_x_ = 1.205 Mg m^−^^3^
Monoclinic, *C*2/*c*	Mo *K*α radiation λ = 0.71073 Å
Hall symbol: -C 2yc	Cell parameters from 5155 reflections
*a* = 23.9157 (4) Å	θ = 1.7–30.0º
*b* = 6.2788 (1) Å	µ = 0.09 mm^−^^1^
*c* = 24.1224 (4) Å	*T* = 100.0 (1) K
β = 101.971 (1)º	Block, colourless
*V* = 3543.49 (10) Å^3^	0.49 × 0.18 × 0.16 mm
*Z* = 8	

### Data collection

**Table:**

Bruker SMART APEXII CCD area-detector diffractometer	5155 independent reflections
Radiation source: fine-focus sealed tube	3632 reflections with *I* > 2σ(*I*)
Monochromator: graphite	*R*_int_ = 0.035
Detector resolution: 8.33 pixels mm^-1^	θ_max_ = 30.0º
*T* = 100.0(1) K	θ_min_ = 1.7º
ω scans	*h* = −33→33
Absorption correction: multi-scan(SADABS; Bruker, 2005)	*k* = −8→8
*T*_min_ = 0.958, *T*_max_ = 0.986	*l* = −33→33
21759 measured reflections	

### Refinement

**Table:**

Refinement on *F*^2^	Secondary atom site location: difference Fourier map
Least-squares matrix: full	Hydrogen site location: inferred from neighbouring sites
*R*[*F*^2^ > 2σ(*F*^2^)] = 0.048	H-atom parameters constrained
*wR*(*F*^2^) = 0.125	*w* = 1/[σ^2^(*F*_o_^2^) + (0.0546*P*)^2^ + 0.7489*P*] where *P* = (*F*_o_^2^ + 2*F*_c_^2^)/3
*S* = 1.10	(Δ/σ)_max_ = 0.001
5155 reflections	Δρ_max_ = 0.30 e Å^−^^3^
213 parameters	Δρ_min_ = −0.27 e Å^−^^3^
Primary atom site location: structure-invariant direct methods	Extinction correction: none

### Special details

**Table:**

Experimental. The data was collected with the Oxford Cyrosystem Cobra low-temperature attachment.
Geometry. All e.s.d.'s (except the e.s.d. in the dihedral angle between two l.s. planes) are estimated using the full covariance matrix. The cell e.s.d.'s are taken into account individually in the estimation of e.s.d.'s in distances, angles and torsion angles; correlations between e.s.d.'s in cell parameters are only used when they are defined by crystal symmetry. An approximate (isotropic) treatment of cell e.s.d.'s is used for estimating e.s.d.'s involving l.s. planes.
Refinement. Refinement of *F*^2^ against ALL reflections. The weighted *R*-factor *wR* and goodness of fit *S* are based on *F*^2^, conventional *R*-factors *R* are based on *F*, with *F* set to zero for negative *F*^2^. The threshold expression of *F*^2^ > σ(*F*^2^) is used only for calculating *R*-factors(gt) *etc*. and is not relevant to the choice of reflections for refinement. *R*-factors based on *F*^2^ are statistically about twice as large as those based on *F*, and *R*- factors based on ALL data will be even larger.

### Fractional atomic coordinates and isotropic or equivalent isotropic displacement parameters (Å^2^)

**Table:**

	*x*	*y*	*z*	*U*_iso_*/*U*_eq_	
O1	0.74746 (4)	1.13213 (14)	0.78815 (4)	0.0273 (2)	
O2	0.62826 (4)	1.08149 (14)	0.74372 (3)	0.0269 (2)	
O3	0.58372 (4)	0.94891 (18)	0.65973 (4)	0.0389 (3)	
O4	0.55809 (4)	1.25800 (16)	0.69566 (4)	0.0339 (2)	
O5	0.63462 (4)	0.40108 (16)	1.02990 (4)	0.0344 (2)	
N1	0.73427 (4)	0.81417 (16)	0.83037 (4)	0.0214 (2)	
C1	0.72075 (5)	0.9674 (2)	0.79119 (5)	0.0210 (2)	
C2	0.66622 (5)	0.90078 (19)	0.75060 (5)	0.0215 (2)	
H2	0.6747	0.8586	0.7132	0.026*	
C3	0.64411 (5)	0.7111 (2)	0.77840 (5)	0.0264 (3)	
H3A	0.6300	0.5991	0.7501	0.032*	
H3B	0.6125	0.7541	0.7968	0.032*	
C4	0.69576 (5)	0.6296 (2)	0.82289 (5)	0.0212 (2)	
H4	0.7142	0.5077	0.8068	0.025*	
C5	0.68121 (5)	0.56408 (19)	0.87850 (5)	0.0207 (2)	
C6	0.69206 (5)	0.3607 (2)	0.89980 (5)	0.0237 (3)	
H6	0.7095	0.2605	0.8792	0.028*	
C7	0.67799 (5)	0.2985 (2)	0.95081 (5)	0.0261 (3)	
H7	0.6861	0.1583	0.9649	0.031*	
C8	0.65208 (5)	0.4434 (2)	0.98043 (5)	0.0260 (3)	
C9	0.64158 (6)	0.6501 (2)	0.96005 (5)	0.0301 (3)	
H9	0.6243	0.7505	0.9807	0.036*	
C10	0.65630 (5)	0.7092 (2)	0.90992 (5)	0.0273 (3)	
H10	0.6494	0.8510	0.8965	0.033*	
C11	0.78953 (5)	0.8054 (2)	0.86899 (6)	0.0310 (3)	
H11A	0.8114	0.9339	0.8644	0.046*	
H11B	0.8105	0.6795	0.8605	0.046*	
H11C	0.7840	0.7970	0.9081	0.046*	
C12	0.58881 (5)	1.0836 (2)	0.69533 (5)	0.0235 (3)	
C13	0.50992 (5)	1.3048 (2)	0.64753 (6)	0.0335 (3)	
C14	0.48639 (8)	1.5091 (3)	0.66671 (10)	0.0726 (7)	
H14A	0.4732	1.4835	0.7020	0.109*	
H14B	0.4543	1.5585	0.6374	0.109*	
H14C	0.5164	1.6179	0.6732	0.109*	
C15	0.53223 (8)	1.3349 (4)	0.59443 (9)	0.0818 (8)	
H15A	0.5462	1.1985	0.5830	0.123*	
H15B	0.5636	1.4383	0.6014	0.123*	
H15C	0.5015	1.3878	0.5642	0.123*	
C16	0.46593 (6)	1.1316 (3)	0.64296 (8)	0.0547 (5)	
H16A	0.4571	1.1058	0.6803	0.082*	
H16B	0.4808	1.0008	0.6293	0.082*	
H16C	0.4311	1.1753	0.6163	0.082*	
C17	0.64211 (8)	0.1904 (3)	1.05145 (7)	0.0444 (4)	
H17A	0.6283	0.1816	1.0869	0.067*	
H17B	0.6828	0.1530	1.0586	0.067*	
H17C	0.6204	0.0913	1.0237	0.067*	

### Atomic displacement parameters (Å^2^)

**Table:**

	*U*^11^	*U*^22^	*U*^33^	*U*^12^	*U*^13^	*U*^23^
O1	0.0283 (5)	0.0219 (5)	0.0342 (5)	−0.0031 (4)	0.0121 (4)	0.0018 (4)
O2	0.0266 (4)	0.0269 (5)	0.0260 (4)	0.0102 (4)	0.0026 (3)	−0.0026 (4)
O3	0.0367 (5)	0.0479 (7)	0.0282 (5)	0.0156 (5)	−0.0021 (4)	−0.0096 (5)
O4	0.0270 (5)	0.0296 (5)	0.0414 (5)	0.0104 (4)	−0.0019 (4)	−0.0008 (4)
O5	0.0511 (6)	0.0321 (6)	0.0239 (4)	−0.0066 (5)	0.0167 (4)	−0.0021 (4)
N1	0.0198 (5)	0.0218 (5)	0.0217 (5)	−0.0030 (4)	0.0023 (4)	0.0007 (4)
C1	0.0222 (5)	0.0204 (6)	0.0224 (6)	0.0020 (5)	0.0098 (4)	−0.0017 (5)
C2	0.0228 (6)	0.0201 (6)	0.0216 (5)	0.0064 (5)	0.0046 (4)	−0.0001 (5)
C3	0.0241 (6)	0.0279 (7)	0.0248 (6)	−0.0042 (5)	−0.0002 (5)	0.0015 (5)
C4	0.0234 (6)	0.0180 (6)	0.0222 (6)	−0.0025 (5)	0.0050 (4)	−0.0011 (5)
C5	0.0202 (5)	0.0207 (6)	0.0210 (5)	−0.0027 (5)	0.0037 (4)	−0.0009 (5)
C6	0.0260 (6)	0.0212 (6)	0.0250 (6)	0.0007 (5)	0.0075 (5)	−0.0001 (5)
C7	0.0316 (6)	0.0206 (6)	0.0262 (6)	−0.0006 (5)	0.0064 (5)	0.0019 (5)
C8	0.0294 (6)	0.0281 (7)	0.0209 (6)	−0.0071 (5)	0.0060 (5)	−0.0031 (5)
C9	0.0389 (7)	0.0252 (7)	0.0287 (6)	−0.0007 (6)	0.0128 (5)	−0.0065 (6)
C10	0.0347 (7)	0.0208 (6)	0.0272 (6)	0.0004 (5)	0.0083 (5)	−0.0003 (5)
C11	0.0228 (6)	0.0358 (8)	0.0312 (7)	−0.0027 (6)	−0.0014 (5)	0.0025 (6)
C12	0.0206 (5)	0.0274 (7)	0.0240 (6)	0.0037 (5)	0.0083 (5)	0.0038 (5)
C13	0.0204 (6)	0.0369 (8)	0.0409 (8)	0.0074 (6)	0.0011 (5)	0.0119 (6)
C14	0.0545 (11)	0.0502 (12)	0.0977 (16)	0.0303 (10)	−0.0199 (11)	−0.0041 (11)
C15	0.0520 (10)	0.134 (2)	0.0651 (12)	0.0401 (13)	0.0254 (9)	0.0665 (14)
C16	0.0263 (7)	0.0538 (11)	0.0761 (12)	−0.0002 (7)	−0.0078 (7)	0.0168 (10)
C17	0.0646 (10)	0.0396 (9)	0.0351 (8)	−0.0043 (8)	0.0242 (7)	0.0091 (7)

### Geometric parameters (Å, °)

**Table:**

O1—C1	1.2257 (15)	C7—H7	0.95
O2—C12	1.3403 (14)	C8—C9	1.3917 (19)
O2—C2	1.4411 (14)	C9—C10	1.3788 (18)
O3—C12	1.1934 (15)	C9—H9	0.95
O4—C12	1.3195 (15)	C10—H10	0.95
O4—C13	1.4854 (15)	C11—H11A	0.98
O5—C8	1.3694 (15)	C11—H11B	0.98
O5—C17	1.4188 (18)	C11—H11C	0.98
N1—C1	1.3404 (15)	C13—C15	1.498 (2)
N1—C11	1.4516 (15)	C13—C16	1.501 (2)
N1—C4	1.4679 (15)	C13—C14	1.512 (2)
C1—C2	1.5182 (16)	C14—H14A	0.98
C2—C3	1.5150 (18)	C14—H14B	0.98
C2—H2	1.00	C14—H14C	0.98
C3—C4	1.5459 (16)	C15—H15A	0.98
C3—H3A	0.99	C15—H15B	0.98
C3—H3B	0.99	C15—H15C	0.98
C4—C5	1.5112 (16)	C16—H16A	0.98
C4—H4	1.00	C16—H16B	0.98
C5—C6	1.3807 (17)	C16—H16C	0.98
C5—C10	1.3951 (17)	C17—H17A	0.98
C6—C7	1.3970 (17)	C17—H17B	0.98
C6—H6	0.95	C17—H17C	0.98
C7—C8	1.3807 (18)		
			
C12—O2—C2	114.90 (9)	C9—C10—C5	121.08 (12)
C12—O4—C13	120.12 (11)	C9—C10—H10	119.5
C8—O5—C17	117.54 (11)	C5—C10—H10	119.5
C1—N1—C11	122.27 (10)	N1—C11—H11A	109.5
C1—N1—C4	115.26 (9)	N1—C11—H11B	109.5
C11—N1—C4	120.90 (10)	H11A—C11—H11B	109.5
O1—C1—N1	126.54 (11)	N1—C11—H11C	109.5
O1—C1—C2	125.62 (11)	H11A—C11—H11C	109.5
N1—C1—C2	107.83 (10)	H11B—C11—H11C	109.5
O2—C2—C3	113.61 (10)	O3—C12—O4	128.22 (11)
O2—C2—C1	107.08 (9)	O3—C12—O2	124.61 (11)
C3—C2—C1	105.26 (9)	O4—C12—O2	107.17 (10)
O2—C2—H2	110.2	O4—C13—C15	109.70 (11)
C3—C2—H2	110.2	O4—C13—C16	109.42 (12)
C1—C2—H2	110.2	C15—C13—C16	113.41 (17)
C2—C3—C4	105.40 (9)	O4—C13—C14	101.90 (12)
C2—C3—H3A	110.7	C15—C13—C14	112.05 (16)
C4—C3—H3A	110.7	C16—C13—C14	109.72 (14)
C2—C3—H3B	110.7	C13—C14—H14A	109.5
C4—C3—H3B	110.7	C13—C14—H14B	109.5
H3A—C3—H3B	108.8	H14A—C14—H14B	109.5
N1—C4—C5	111.08 (9)	C13—C14—H14C	109.5
N1—C4—C3	102.39 (10)	H14A—C14—H14C	109.5
C5—C4—C3	114.10 (10)	H14B—C14—H14C	109.5
N1—C4—H4	109.7	C13—C15—H15A	109.5
C5—C4—H4	109.7	C13—C15—H15B	109.5
C3—C4—H4	109.7	H15A—C15—H15B	109.5
C6—C5—C10	118.05 (11)	C13—C15—H15C	109.5
C6—C5—C4	121.46 (11)	H15A—C15—H15C	109.5
C10—C5—C4	120.49 (11)	H15B—C15—H15C	109.5
C5—C6—C7	121.68 (12)	C13—C16—H16A	109.5
C5—C6—H6	119.2	C13—C16—H16B	109.5
C7—C6—H6	119.2	H16A—C16—H16B	109.5
C8—C7—C6	119.19 (12)	C13—C16—H16C	109.5
C8—C7—H7	120.4	H16A—C16—H16C	109.5
C6—C7—H7	120.4	H16B—C16—H16C	109.5
O5—C8—C7	125.01 (12)	O5—C17—H17A	109.5
O5—C8—C9	115.06 (12)	O5—C17—H17B	109.5
C7—C8—C9	119.93 (12)	H17A—C17—H17B	109.5
C10—C9—C8	120.04 (12)	O5—C17—H17C	109.5
C10—C9—H9	120.0	H17A—C17—H17C	109.5
C8—C9—H9	120.0	H17B—C17—H17C	109.5
			
C11—N1—C1—O1	−12.17 (19)	C3—C4—C5—C10	−58.06 (15)
C4—N1—C1—O1	−177.97 (11)	C10—C5—C6—C7	0.94 (18)
C11—N1—C1—C2	167.57 (11)	C4—C5—C6—C7	−179.13 (11)
C4—N1—C1—C2	1.77 (13)	C5—C6—C7—C8	0.63 (18)
C12—O2—C2—C3	−88.44 (12)	C17—O5—C8—C7	−2.06 (19)
C12—O2—C2—C1	155.79 (10)	C17—O5—C8—C9	177.48 (13)
O1—C1—C2—O2	−48.13 (15)	C6—C7—C8—O5	177.96 (11)
N1—C1—C2—O2	132.13 (10)	C6—C7—C8—C9	−1.56 (19)
O1—C1—C2—C3	−169.34 (11)	O5—C8—C9—C10	−178.65 (12)
N1—C1—C2—C3	10.92 (12)	C7—C8—C9—C10	0.9 (2)
O2—C2—C3—C4	−135.30 (10)	C8—C9—C10—C5	0.7 (2)
C1—C2—C3—C4	−18.46 (12)	C6—C5—C10—C9	−1.61 (18)
C1—N1—C4—C5	−135.47 (11)	C4—C5—C10—C9	178.45 (11)
C11—N1—C4—C5	58.52 (14)	C13—O4—C12—O3	0.5 (2)
C1—N1—C4—C3	−13.27 (13)	C13—O4—C12—O2	−179.21 (10)
C11—N1—C4—C3	−179.29 (11)	C2—O2—C12—O3	0.52 (18)
C2—C3—C4—N1	18.89 (12)	C2—O2—C12—O4	−179.77 (9)
C2—C3—C4—C5	139.01 (11)	C12—O4—C13—C15	−63.23 (18)
N1—C4—C5—C6	−122.88 (12)	C12—O4—C13—C16	61.79 (16)
C3—C4—C5—C6	122.01 (12)	C12—O4—C13—C14	177.89 (14)
N1—C4—C5—C10	57.05 (14)		

### Hydrogen-bond geometry (Å, °)

**Table:**

*D*—H···*A*	*D*—H	H···*A*	*D*···*A*	*D*—H···*A*
C2—H2···O1^i^	1.00	2.35	2.9633 (15)	119
C4—H4···O1^ii^	1.00	2.56	3.5238 (15)	162
C11—H11A···O1	0.98	2.47	2.8652 (16)	104
C15—H15A···O3	0.98	2.45	3.011 (3)	116
C16—H16B···O3	0.98	2.44	2.9904 (19)	115
C17—H17A···O3^iii^	0.98	2.38	3.324 (2)	161

Symmetry codes: (i) −*x*+3/2, *y*−1/2, −*z*+3/2; (ii) *x*, *y*−1, *z*;  (iii) *x*, −*y*+1, *z*+1/2.
